# Dielectric Fluids for Power Transformers with Special Emphasis on Biodegradable Nanofluids

**DOI:** 10.3390/nano11112885

**Published:** 2021-10-28

**Authors:** Miloš Šárpataky, Juraj Kurimský, Michal Rajňák

**Affiliations:** 1 Faculty of Electrical Engineering and Informatics, Technical University of Košice, Letná 9, Košice 04200, Slovakia; milos.sarpataky@tuke.sk (M.Š.); michal.rajnak@saske.sk (M.R.); 2 Institute of Experimental Physics SAS, Watsonova 47, Košice 04001, Slovakia

**Keywords:** nanofluid, insulation oils, biodegradable fluids, nanoparticles, stability

## Abstract

This review is focused on the research of dielectric fluids, especially commonly used power transformer oils enhanced by nanoparticles, i.e., nanofluids. There are differences between various combinations of base fluids and nanoparticles prepared in different ways. The main goal of this review was to present recent research in this field sorted by the used nanoparticles. Nanofluids based on mineral oils, natural, or synthetic esters were investigated in terms of the nature of nanoparticles, particularly Al_2_O_3_, TiO_2_, Fe_2_O_3_, Fe_3_O_4_, graphene, fullerene, and others. The combinations of environmentally friendly oils and nanoparticles were presented. Finally, the article focused on the description of current dielectric fluids usable in power transformers and the possibilities of improving new and existing fluids with nanoparticles, especially their physical, dielectric, and chemical properties, but with regard to environmental aspects.

## 1. Introduction

**Figure 1:**
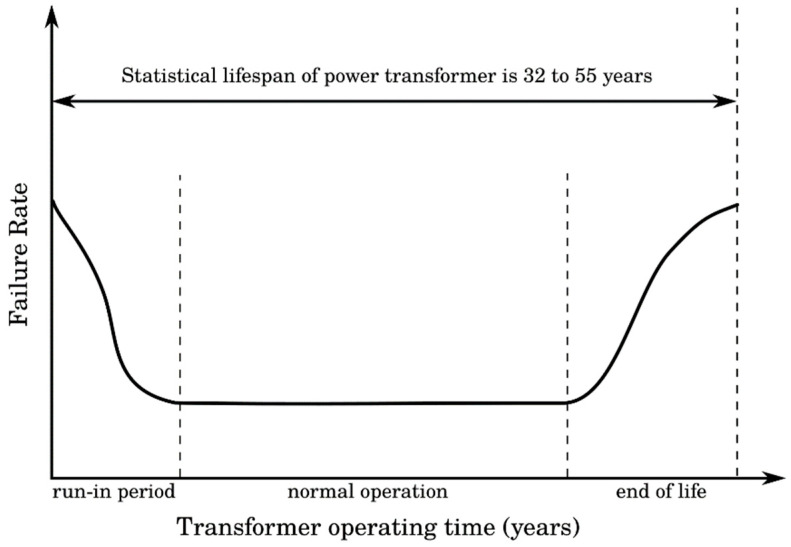
General “bathtub” pattern as the failure rate of power transformers.

According to the sustainable development, many challenges must be solved in the world’s industry, especially in power engineering. This important part of the industry faces great pressure to improve technologies to ensure the supply of electricity, with having regard for all strict regulations and environmental protection. The greatest attention is paid to the production and consumption of electricity, but also many other parts of this industry, such as transmission and distribution of electricity, are key to fulfil goals and needs regarding the sustainable development [1]. One of the affected parts of this industry facing new challenges and modifications is the insulation of power transformers, especially insulating oils. Power transformers, as one of the most important devices in power engineering, are used for the transformation of voltage levels or for galvanic separation of electrical systems with the same voltage. To secure the operation of the power transformer, heat transfer and insulation of different parts have the highest priority. The most common failures in transformers are related to the quality of materials, random defects, and age. The age-related failures have a typical “bathtub” pattern depicted in Figure 1 that shows the probability of failure related to transformer operating time. In the early years of service, there is a higher number of failures due to infant mortality. A low number of failures with constant failure rate lasts until the age when power transformers are affected for example by moisture or contamination by different kinds of insoluble particles and gases that cause a decrease in internal dielectric strength [2]. The biggest number of failures are located in windings, bushings, tank, or tap chargers, and these failures are mainly electrical, lighting, insulation, and connection caused [3,4]. The lifespan of a power transformer is around 40 years, more precisely from 32 to 55 years with a standard deviation of 8 years, according to the design, loading, insulation, humidity, and working temperature [5,6]. According to [7], the lifespan of a transformer that failed because of the insulation is 17.8 years, which is only half of the estimated lifetime. According to [8], transformer oil lifetime prediction is 20.55 years, but in bad conditions, it can be lowered to 5.5 years. This information confirms the importance of progressive research on insulating fluids, their enhancement, analysis, and substitution.

Throughout history, there have been different kinds of insulating fluids used in power transformers. Rafiq et al. in [9] offered an eloquent historical review. In short, the origin of this field of study is in the year 1890 when the first oil-immersed transformer was developed and filled with petroleum-based liquid, so-called paraffin oil [10]. This oil had a low viscosity and outstanding insulation properties, but it was replaced after the year 1925 when the pour point was determined as too high and due to the insoluble particles (the result of oxidation) that affected its heat transfer and finally resulted in a shorter lifespan. For a few years, naphthenic oils replaced paraffin oils because of a higher pour point and better resistance to oxidation. However, this oil was very flammable and had environmental contaminant status, so it had to be replaced, too. PCBs oils (polychlorinated biphenyls) used for 40 years, till 1970, met the requirements for good liquid insulation, but the fact that they are toxic, changed its status to environmentally unacceptable. In year 1978 PCBs were banned [9,11].

The followers of PCB oils were mineral oils and silicone fluids as a new alternative, and their usage continues even today. The development of insulating fluids, later focused more on ecological aspects like biodegradability, led to the discovery of natural and synthetic esters as environmentally friendly fluids. All insulating fluids have to meet certain requirements and the most important features for categorization and rating of quality are thermal conduction, viscosity, fire point, biodegradability, dissipation factor breakdown voltage, and resistivity of fluids [12]. Moreover, the presence of moisture in liquid affects the mentioned properties, so it is very important to find out the moisture content and how water in each state influences the properties of different fluids. For example, in [13], there is a description of the influence of saturated moisture in transformer oils on the breakdown voltage, finding that 20% moisture saturation before and after the ageing process did not affect natural or synthetic esters, but a value (breakdown voltage) of N3X (high-grade mineral oil Nynas Nytro 3000X) was halved.

The most frequently used insulation fluid in power transformers is mineral oil due to its good dielectric and thermal properties. However, due to their environmental footprint, they are currently considered unsatisfactory. The tendency of actual research is to replace mineral oils with natural (vegetable oils) or synthetic esters as more environmentally friendly alternatives [14]. If one compares the biodegradability of mineral oil and natural or synthetic esters, it is obvious that the esters are much more environmentally safer with biodegradability more than 80%, while mineral oils reach the values of less than 30% [15]. Many studies deal with the comparison of environmentally friendly liquids with mineral oils, for example [9,12,16,17]. To sum up, the biggest advantage of mineral oils is a low viscosity, low dissipation factor, and high resistivity and breakdown voltage that shows its predestination for high voltage equipment. On the other hand, the weakest point is the safety, because of its low fire and flame point and low biodegradability that can cause environmental damage and problems during liquidation of used oils.

Recent research has revealed that there is a possibility to improve the heat transfer and dielectric properties of the insulating liquids by introducing various nanoparticles. A mixture of nanoparticles and a base fluid is called a nanofluid. Nanofluids are one of the possible options as a substitution for the currently most used insulating fluids for high voltage equipment [9]. Power engineering, as one of the most important industries, is heading to increase the transmission voltage to keep the distribution of electricity sufficient and reliable, so the need for a new and more effective insulation is justified [18]. Furthermore, high voltage substations and equipment are of large area and because of that, there is a need to smaller the size of this equipment that goes hand in hand with a more efficient insulation and heat transfer medium. Nanofluids are a possible option for reducing the size of equipment because of their better resistivity to overvoltages, and degradation caused by humidity in comparison to base fluids [19]. Moreover, the fact that nanoparticles may improve the heat transfer properties confirm the possibility to construct smaller high voltage equipment filled with oil. A prototype of a ferrofluid-cooled transformer with reduced sizes is published in [20]. Concerning the environmental aspects, the mixture of environmentally friendly oils and nanoparticles may be a good option for maintaining the sustainable development and quality requirements [21].

## 2. Different Kinds of Base Fluids

Generally, the most used transformer oils are mineral oils (MO). The reason for their usage is the fact they have excellent performance in practice and they are very reliable with a long tradition. Mineral oil is created from crude oils (fossil fuels) that contain hydrocarbon compounds of different bonds [16,22]. It is a translucent liquid “composed of hydrocarbons, among which are straight-chain alkanes, branched alkanes, cyclic paraffin and aromatic hydrocarbons” [23]. The disadvantage of paraffinic oils is their high pour point that must be treated by additives to reach the required values. The difference between them is not very significant and they are classified as very naphthenic if there is a minimum of 50% of the naphthenic structure, intermediate—containing 44–50% of the naphthenic structure, and below 44% of the naphthenic structure in MO is considered to be paraffinic [23].

Mineral oil as a potential environmental contaminator has low biodegradability that is a possible critical issue for its usage in future. Mineral oil has a relatively low fire and flash point that may be a safety issue for the environment and people and it could cause serious environmental damage after ignition and leakage from the power transformer, finally resulting in an ecological problem for the affected region [17,24]. However, Table 1 shows high values of viscosity and dissipation factor as important properties for insulating oil, concretely viscosity for heat transfer and dissipation factor for economic issues (power loss). To sum up, MO is an excellent insulating fluid tested in practice, but it conflicts with the sustainable development.

The most convincing difference between MO and silicone fluids (SF) is flammability. SF have significantly higher fire and flame points with similar values of insulation properties as MO. A big contrast in safety between SF and MO can be seen from Table 1, pointing out a difference in fire point up to 240 °C. SF is chemically known as polydimethylsiloxane and it is not frequently used mainly because of a higher price than MO, and because of biodegradability, which is at the lowest level in comparison with other base fluids that makes the biggest environmental impact [16,25].

A major component of synthetic esters (SE) is pentaerythritol tetra ester developed from chemicals with biodegradability at a level from 80 to 89%. Firstly, SE as an insulation liquid in power transformer was used in 1976. Synthetic esters are mostly used in special and distribution transformers with a relatively low level of voltage [9,21]. Comparison between SE and MO in Table 1 indicates that the properties are comparable and, in some cases, even better for SE. The most noticeable disadvantage of SE is the high dissipation factor [26]. The most significant commercial SE are MIDEL 7131, NYCODIEL 1244, BecFluid 9902, ECO-FR PDS, Naturelle Transformer Oil S4, Envirotemp 200 [16].

Natural esters (NE) are the best option as environmentally friendly insulating fluids for high voltage equipment. Commercial availability of natural esters has a rising tendency that makes it one of the most likely options for the power transformers refilling. NE is defined as a refined vegetable oil or a plant-based ester derived from crops. For the extraction of oil, seeds or other parts of a suitable plant are used [9]. Biodegradability at a level from 97 to 99% makes the most significant difference in comparison to other insulating oils. This high level of biodegradability is connected with the safety of this insulating liquid mainly in the case of a possible leakage. The advantage of high fire and flame points is confirmed by statistics from 2014 when there was no reported fire or environmental issue in over 600,000 transformers using NE [27]. Disadvantages of NE are relatively high pour point that is in some cases −10 °C and high dissipation factor according to the type of NE up to 0.4 [28]. The most significant commercial NE are BIOTEMP, Envirotemp FR3, ambient insulating fluid, ambient prime insulating fluid, NeuGen 1540 [16]. SE have a more stable chemical structure than NE and it results in a better oxidation stability. NE have the worst oxidation stability among the mentioned base fluids [24]. However, the addition of nanoparticles into a base fluid may enhance the electrical properties and NE as an environmentally friendly fluid may be a suitable option for the refinement. Moreover, the enhanced properties may assure the ecologically friendly insulation fluid that could meet the requirements for sustainable development as the future most widely used insulation liquid.

Table 1 compares various properties of the mentioned base fluids. The range of values is connected with different references with various experimental results.

**Table 1:** Different properties of base fluids.

Properties	MO	SF	NE	SE	Reference
Biodegradability in 21 days [%]	<30	very low	97–99	80–89	[9,16,17]
Viscosity at 40 °C [mm^2^/s]	3–16	35–40	16–37	14–30	[14,26]
Flash point [°C]	100–175	300–310	250–316	260–343	[16,17,26]
Fire point [°C]	110–185	330–350	300–370	300–322	[16,17,26]
Pour point [°C]	−30 to −63	−50 to −60	−10 to −33	−40 to −60	[16,17,26]
AC breakdown voltage [kV]	30–85	35–60	49–97	43–75	[16,17,26,29]
Dissipation factor at 90 °C [–]	<0.001	0.0016	0.0001–0.009	0.001–0.03	[13,16]
Resistivity at 90 °C [GΩm]	10^13^–10^15^	10^14^	10^13^–10^14^	10^13^	[9,16]

## 3. Nanofluids

There are many different mixtures tested in research history with different insulation fluids and nanoparticles, but there are also different ways of preparing nanofluids categorized in a one-step process and a two-step process. According to [9], the origin of the word “nanofluid” is connected with the work by Choi et al. back in 1995, where they researched enhancement of thermal conductivity of fluids mixed with nanoparticles, determined the direction of further research, described the theoretical study of these fluids, and set potential benefits of nanofluids [30]. From that point, the number of scientific papers that deal with nanofluids has been exponentially increasing. Till 2008, publications on the topic of nanofluids did not exceed number 100 throughout the year. Since 2008 there has been a more significant increase of scientific papers year by year [31].

**Figure 2:**
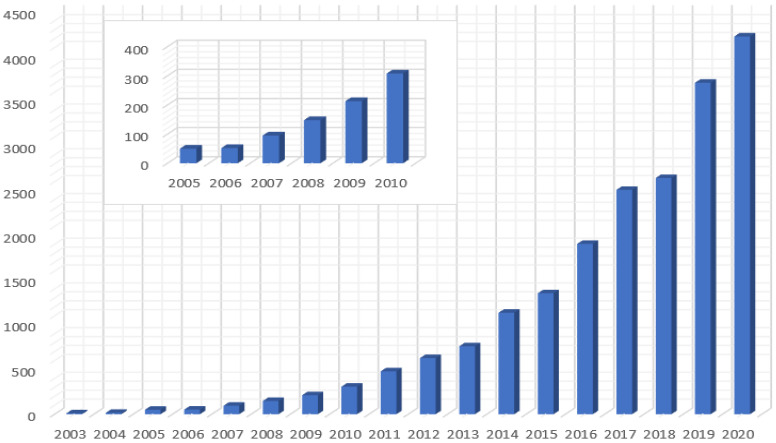
Number of publications on topic “nanofluid”.

Figure 2 shows the number of publications on the web of science core collection year by year made by basic search of word “nanofluid”. The biggest growth of scientific papers was in recent years when the topic of enhancement of heat transfer and insulating fluid is more discussed, because of increasing demand on life and industry. More than 36% of these papers are in the category of thermodynamics, 22% mechanics and 21% belong to mechanical engineering. Of course, the paper may be in more categories at once. The highest increase of papers was in 2019 when the number of scientific papers dealt with nanofluids was 3707 in comparison with 2642 papers in 2018. Last year, there have been more than 4200 papers published mostly in the categories of thermodynamics, mechanics, and physical chemistry.

### 3.1. One-Step Method

In a one-step process of preparation of nanofluids, the formation and dispersion of nanoparticles are simultaneous [32]. This method avoids the process of transportation, storage, drying, and dispersion of nanoparticles to decrease a measure of agglomeration of nanoparticles and to increase the stability of nanofluids [33,34,35]. One of the one-step preparation methods is a vapor deposition. This method was patented by Choi and Eastman in 2001 [36]. The principle consists in the formation of a thin layer of a base fluid on a vessel wall caused by a centrifugal force of a rotating disk. The material is then heated and evaporated in the vessel filled with an inert gas at a low pressure. The creation of nanofluid is finished when the raw material vapors condense by interacting with the thin film of swirling water and finally settle in the base fluid [32,36]. The second possible one-step method is the laser ablation where a highly concentrated laser beam is used for dispersion of nanoparticles from the surface of a material immersed in a base fluid. Important properties are the intensity and the wavelength of the laser beam [37,38]. There are more possible preparation methods using the one-step process such as the submerged arc method, precipitation (ion exchange) method, chemical reduction method, emulsion polymerization, sol-gel (hydrolysis) method, or microwave-assisted reaction [32,39,40]. The submerged arc nanoparticle synthesis system is an efficient one-step method to prepare nanofluids based on dielectric liquids containing copper nanoparticles [41,42].

### 3.2. Two-Step Method

**Figure 3:**
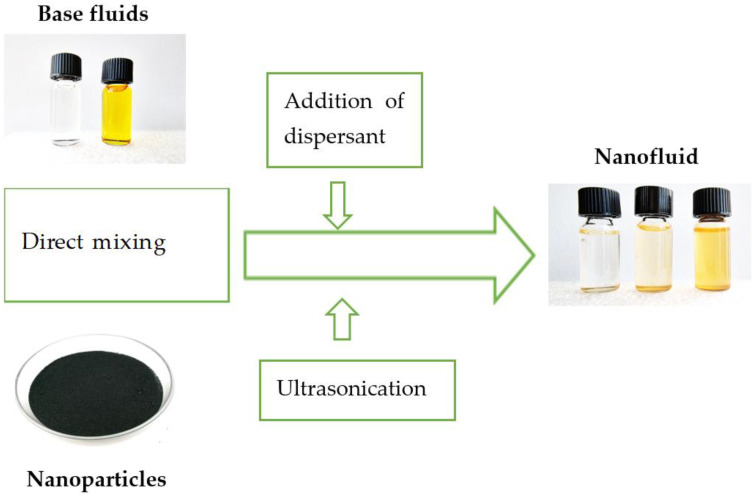
A simple illustration of the two-step method of preparing nanofluids. Inset photographs are from the authors archive.

In the first phase of the two-step method, nanoparticles (nanorods, nanofibers, or nanotubes) are first prepared by sol-gel method, hydrothermal synthesis, or by other techniques [32]. The sol-gel method is used for nanoparticles with high surface area and provides effective control over the texture and surface properties of nanoparticles. There are five main steps of this method starting with hydrolysis and then polycondensation, ageing, drying, and thermal decomposition [32,40,43]. According to [44,45], hydrothermal synthesis refers to the “heterogeneous reactions for synthesizing inorganic materials in aqueous media above ambient temperature and pressure” and the advantage of this method is the low energy consumption, the low-temperature processes, and the environmental impact. However, the high price of the needed autoclaves may be considered as a potential drawback [32]. The second step of the two-step method is the preparation of nanofluids using mostly ultrasonication (the bath and probe ultrasonication are of remarkably different effectivity), magnetic stirring, adjusting pH value (addition of a dispersant in Figure 3), or a combination of these processes [32]. Ultrasonication enhances performance, stability, thermophysical properties and prevents aggregation and sedimentation of nanoparticles in nanofluid, but the ideal duration of sonication is the point of research of many authors [46,47,48,49,50]. Magnetic stirring is used for dispersion of nanoparticles with a low concentration by the stirring action made by a stir bar that spins very quickly because of a rotating magnetic field created by the set of rotating magnets or electromagnets [32,51,52].

Selecting a particular method, additives and timing within the applied methods are very important to make a nanofluid with excellent properties without sedimentation and agglomeration of nanoparticles. A more accurate description of preparation methods of eleven different nanoparticles is in the reference [53].

To compare the two preparation methods, one can state that the two-step method is more economic in production of nanofluids in a larger scale and it is used most widely. However, without modifying the nanoparticle surface, the tendency to agglomerate before adding to the base liquid is considered as a major disadvantage of the two-step method. On the other hand, the one-step method cannot be effectively used to synthesize nanofluids in a large scale, but this method can yield uniformly and stably dispersed nanoparticles.

### 3.3. Stability of Nanofluids

The stability of nanofluids is of crucial importance because the sedimentation and agglomeration of nanoparticles in the base fluid cause deterioration of thermophysical properties, mainly a decrease of thermal conductivity and increase of viscosity [54,55,56]. Because of these facts, examination of stability is an important part of research and there are several methods to analyze the stability of synthesized nanofluids. The most applied methods to study the stability are a zeta potential test, a sedimentation method (photograph capturing method), ultraviolet-visible spectrophotometer and dynamic light scattering [56,57].

#### 3.3.1. Zeta Potential Method

The Zeta potential (ζ-potential) method is the most used method to examine the stability of nanofluids. It can be defined as the potential difference between the stationary layer of base fluid which is attached to nanoparticles and the surface of nanoparticles [54]. It indicates the degree of repulsion between charged particles in the fluid and it can be calculated by the Helmholtz–Smoluchowski equation [58,59]:

ζ = *μU*/*ε* (1)

where *U* is electrophoretic mobility, *µ* is viscosity, and *ε* is the dielectric constant of the base liquid. If the value of Zeta potential is over 60 mV, a nanofluid has an excellent stability, between 60 and 40 mV it has a good stability, between 40 and 30 mV the nanofluid is considered as stable, and below 30 mV it is considered as highly agglomerative [57,60,61]. Because of repulsive forces, the Zeta potential can be controlled over pH value [62,63]. A change of pH influences the surface charge on nanoparticles and modifies their interaction behavior [57]. If the pH of the nanofluid has low values, the Zeta potential will be positive. On the other hand, with higher pH values, the Zeta potential will be in negative values. The point when a pH value corresponds to zero Zeta potential is called the isoelectric point, when nanofluids are least stable, so stability rises in the positive or negative direction from that point [57]. Measurements of the Zeta potential are the most often performed by a Zeta Sizer Nano (ZSN) device [60,64,65].

#### 3.3.2. Sedimentation Method (Photograph Capturing Method)

The sedimentation method is the simplest option of measuring the stability of nanofluids qualitatively, by observing photographs taken in different periods [60,66]. Owing to external forces (gravitation), the nanoparticles settle on the bottom of the fluid in a clear glass test tubes that can be observed by comparing photographs taken at different times [67]. In an unstable nanofluid there are three ways of sedimentation. The first one is a dispersed sedimentation where the height of a sediment rises from the bottom. The second one is a flocculated sedimentation where the sedimentation is lowered with time, and the last one is a mixed sedimentation where the behavior of both previous cases is observed at the same time [60,67,68]. The most significant impact on sedimentation has the concentration of nanoparticles and properties of the base fluid [69,70].

#### 3.3.3. Ultraviolet-Visible Spectrophotometer

The ultraviolet-visible spectrophotometer is commonly used for the quantitative characterization of the colloidal stability of the dispersions [56]. One of the major advantages of this method is its suitability for all base fluids because its functioning is about the intensity of the light that becomes different because of lights scattering and absorption when passing through the fluid [57,61,71]. According to [57,71] and [72], the range of UV–visible spectrophotometer is from 200 to 900 nm wavelengths and basically, it measures various dispersions in the fluid. The stability is determined by the dispersion of nanoparticles in different time results [73].

#### 3.3.4. Dynamic Light Scattering

Dynamic light scattering is a suitable method for measuring mainly spherical particles and the most significant advantage is that this method does not need drying of the dispersion (some dispersants are difficult to remove) [74,75]. A simple description of this method is that a source of monochromatic light shines on the sample and a detector collects the scattered light signals [72,76]. There is a need to know the refractive index and viscosity of a measured base liquid, and the measurement output is a signal that shows random changes due to the randomly changing relative position of the particles due to the random Brownian motion. Size as the final output is calculated by the Stokes–Einstein equation [61,62].

### 3.4. Structural Characterization of Nanofluids

The essential parameters determining the physical properties of nanofluids are the nanoparticle size distribution, morphology, crystal structure, and elemental composition. Several techniques can be used to characterize nanoparticles from these points of view. In this chapter we mention just a few techniques.

#### 3.4.1. Transmission Electron Microscopy (TEM) and Scanning Electron Microscopy (SEM)

These methods have evolved over many years into a highly sophisticated instrument and have found different applications across many scientific disciplines, because of their excellent ability to distinguish the shape, size, and distribution of nanoparticles [77,78,79]. In [57], the methodology of transmission electron microscopy is described as: “the electrons shoot through the sample and measures how the electron beam changes as it is scattered in the sample. Scanning electron microscope images the sample surface by scanning it with electron beams in a raster scan pattern. The electrons interact with the sample atoms producing signals that contain information about the sample’s surface topography, composition and other properties”. The disadvantage of this method is that it does not capture the real situation of nanoparticles in nanofluids because there is a need for dried samples prepared in a vacuum oven [61,80].

#### 3.4.2. X-ray-Based Techniques

One of the most extensively applied methods for nanoparticle characterization is X-ray diffraction (XRD) [81]. It provides information on the crystal structure, nature of the phase, crystalline grain size and lattice parameters. For this method, the nanoparticles in powder form are commonly used after drying the colloidal solution. XRD provides statistically representative, volume averaged values. For instance, this method has been applied to determine the average crystallite size of magnetite nanoparticles [82]. Another X-ray-based analytical method to determine the structure of nanoparticles in terms of averaged size or shapes is small angle X-ray scattering (SAXS) [83]. Normally, a transmission mode is used, when the X-ray beam is sent through the nanofluid sample and the average structure of all illuminated particles is measured.

#### 3.4.3. Neutron Scattering Techniques

Analogously to the SAXS method, small angle neutron scattering (SANS) is often used to study the structure of nanofluids, in terms of nanoparticle size and shape distribution, but also to study assembly and alignment of nanoparticles [84]. Among the advantages of neutrons, one can highlight their larger penetration depth and an option of using contrast variation. In this way, different parts of a sample can be selectively viewed via isotopic labelling. This method has been found especially useful in structure research of magnetic nanofluids [85].

#### 3.4.4. Atomic Force Microscopy

Atomic force microscopy (AFM) is a technique capable of providing three-dimensional images of surfaces. It measures the interacting forces between a fine probe and the sample. In this way, individual particles and groups of particles can be resolved and shape and size distribution of nanoparticles can be obtained [86]. AFM can scan the sample under different modes depending on the degree of proximity between the probe and the sample (contact, non-contact, and tapping mode). The tapping mode is the most common when characterizing nanoparticles [87].

Clearly, in order to get the complete picture of an unknown sample, which allows extraction of proper correlations between the structure and the improvement of the base liquid properties, one needs to employ various methods, because their results are complementary.

## 4. Current Experimental Results of Nanofluid Properties Sorted by Used Nanoparticles

This section will describe the latest research on nanofluids and their physical and dielectric properties. Searching for the articles was performed in google scholar database and web of science services by using the keyword “nanofluid” with different specifications as a second word such as “breakdown”, “synthetic ester”, or “biodegradable oils”, while only articles published after the year 2019 have been considered. The following sections are divided according to the nanoparticles used in the reviewed publications. The overview is mainly focused on quantities describing insulation properties, mainly alternating current breakdown voltage (AC-BDV), direct current breakdown voltage (DC-BDV), lighting impulse breakdown voltage (LI-BDV), partial discharge inception voltage (PDIV), and dissipation factor (tanδ).

### 4.1. Al_2_O_3_ Nanoparticles

Al_2_O_3_ nanoparticles are the insulating type of nanoparticles with good thermal and dielectric properties often used in research to improve the properties of transformer oils. The electrical conductivity of these nanoparticles is from 10 to 12 Sm^−1^ and thermal conductivity is around 25.5 Wm^−1^/°C (at 25 °C) that makes these particles suitable for dielectric improvement of base fluids [88].

Jacob et al. [89] measured thermophysical properties of nanofluids with Al_2_O_3_ nanoparticles with different base fluids. Mineral oil and soybean natural ester were filled with nanoparticles (size 60 nm) with concentrations 0.002 wt%, 0.01 wt%, 0.02 wt%, 0.04 wt%, and 0.1 wt%. The optimal stability was at concentration 0.02 wt% for both types of oils, while NE was more stable than MO. The experiment on thermophysical properties compares thermal conductivity and viscosity of the examined oils. The thermal conductivity of unfilled NE was 5.4% higher than that of MO. The values of thermal conductivity of NE and MO were improved at each concentration and the difference at concentration 0.1 wt% was 14.6% comparing NE and MO. The viscosity of NE was four times higher than the viscosity of MO, but after the addition of nanoparticles, the increase in viscosity of NE and MO (at concentration 0.1 wt%) was 14.2% and 29.2%, respectively. According to the thermophysical properties, NE based nanofluid seems to be the better option as a heat transfer medium.

Oparanti et al. [90] measured the viscosity of nanofluid with Al_2_O_3_ nanoparticles. The viscosity was decreased by 11.9% at 40 °C with a concentration of nanoparticles 0.2 wt%. With higher concentrations, there were no significant changes in viscosity. The experimental fluid was palm kernel oil and the size of nanoparticles were 18 nm proved by XRD analysis. The flash and pour points of this nanofluid were improved by 9% and 5%, respectively. As for dielectric properties, the dissipation factor was improved (lowered) by adding the nanoparticles and tanδ decreased with the concentration of nanoparticles. The effect of different base oil with Al_2_O_3_ nanoparticles on dissipation factor were examined by Chakraborty et al. [91] and it may be observed that the presence of nanoparticles in MO increases the value of tanδ that is connected with an increase in power loss and on the other hand, the presence of Al_2_O_3_ nanoparticles in NE (FR3) reduces the value of tanδ. These results manifested that the importance of research on different base fluids with specific nanoparticles is crucial, because of significant differences in results.

#### 4.1.1. AC-Breakdown Voltage

Most of the recent research on nanofluids for power transformers examine dielectric properties, mainly the value of the breakdown voltage. Khaled et al. examine AC-BDV on a nanofluid composed of MO and Al_2_O_3_ nanoparticles of two sizes, 13 and 50 nm [92]. The nanofluid showed maximum enhancement 76.3% of AC-BDV at concentration 0.05 g/L with nanoparticles 13 nm large. With higher concentration up to 0.4 g/L, the values of enhancement fell to 31.4% that is still a significant improvement of insulating oil properties. With a particle size of 50 nm, the maximum improvement was 69.1% at concentration 0.3 g/L and enhancement of more than 36.8% was found for all concentrations of the nanoparticles.

A similar experiment was carried out with synthetic ester MIDEL 7131 by the same authors [93]. All concentrations of Al_2_O_3_ nanoparticles in the base fluid showed enhancement in AC-BDV. The optimal concentrations 0.05 and 0.3 g/L were found for particle sizes 13 and 50 nm, respectively, the same as in the measurements with MO. Particles of size 13 nm improve AC-BDV from 34.7% (0.05 g/L) to 16.5% (0.4 g/L) and 50 nm nanoparticles increased the value from 10.5% (0.05 g/L) to 25.5% (0.3 g/L).

Rafiq et al. [94] prepared a mixture of MO and Al_2_O_3_ nanoparticles with a concentration 0.8 g/L and examined BDVs after ageing. Before thermal ageing, the enhancement of AC-BDV was around 14% and the values of AC-BDV decreased with ageing time for both base fluid and nanofluid. The difference between these two samples dropped after 30 days of ageing to 8%.

Zhang et al. [95] made a mixture of MO and Al_2_O_3_ nanoparticles of size less than 20 nm. Different concentrations of nanoparticles from 0.01 to 0.05 g/L were tested on AC-BDV with different relative humidity from 10% to 80%. All the samples showed a decrease in AC-BDV with the rising level of humidity. However, nanofluids are not as sensitive to humidity as pure MO. The difference in AC-BDV values between a pure oil and nanofluid with the concentration of nanoparticles 0.03 g/L raised from around 8% (at 10% humidity of examined fluids) to 82% (at 80% humidity), thus confirming that nanofluids are less sensitive to moisture. According to [96,97] it is caused by dissolved water bound to the surface of nanoparticles, where some multimolecular water clusters might be broken into single water molecules and might be attached to the surface of some nanoparticles that cause enhancement in AC-BDV in presence of water.

One of the aspects that makes difference in results is the modification of nanoparticles’ surface, which primarily improves the stability of nanofluids. Jacob et al. [98] made MO with Al_2_O_3_ nanoparticles of diameter 60 nm with and without 0.1 wt% of surfactant (oleic acid) at a concentration of nanoparticles 0.1 wt% and 0.03 wt%. Only the samples with the concentration 0.03 wt% with and without surfactant exhibited higher values of AC-BDV than the pure oil. The difference in enhancement was 6.6% and 16.6% for the nanofluid (concentration 0.03 wt%) without and with the oleic acid surfactant, respectively.

Baharuddin et al. [99] examined different concentrations of surfactant (cetyltrimethylammonium bromide (CTAB)), which was added to the mixture of MO and Al_2_O_3_ nanoparticles with a diameter of 13 nm and concentrations 0.1 wt% to 0.5 wt%. The maximum improvement showed the nanofluid with 0.1 wt% of Al_2_O_3_ nanoparticles. Concentrations of CTAB in 0.1 wt% nanofluid ranged from 0.025% to 0.1% and results showed that optimal concentration of surfactant is 0.075%.

Opranati et al. [100] examined the breakdown strength of NE (palm kernel oil) with Al_2_O_3_ nanoparticles. The measured concentrations of the nanofluid were from 0.2 wt% to 1 wt% in the step of 0.2 and the optimal concentration was 0.6 wt% that reached an enhancement of breakdown strength 39.7%. Moreover, each measured concentration showed enhancement from 17% at concentration 0.2 wt% to the mentioned maximum. An overview of the AC-BDV of nanofluids containing Al_2_O_3_ nanoparticles is presented in the following Table 2.

#### 4.1.2. DC-Breakdown Voltage

In the mentioned experiment made by Oparanti et al. [90], the DC breakdown voltage (BDV) was also examined. Enhancement around 38% was observed at concentrations 0.6 wt% and 0.8 wt% in comparison to pure oil, but the mean value of breakdown voltage was 29 kV that makes this combination of NE made of palm kernel oil and Al_2_O_3_ nanoparticles suitable for distribution transformers of relatively low voltage.

Khaled et al. [101] created a nanofluid made of NE (MIDEL 1204) and Al_2_O_3_ nanoparticles of sizes 13 and 50 nm. The maximum enhancement in DC-BDV was approximately 9.2% at concentration 0.3 g/L of nanoparticles 50 nm large. The same concentration of nanoparticles, but with a size of 13 nm, showed a maximum improvement of 7.6%. The nanofluid with 13 nm nanoparticles showed enhancement at concentrations 0.3 and 0.4 g/L and decrement at lower concentrations (0.05 and 0.2 g/L), not more than 2%. The nanoparticles of size 50 nm were more sensitive to concentration and decrements at concentrations 0.05 g/L and 0.4 g/L were 17.2% and 15.7%, respectively. To sum up, the optimal concentration of Al_2_O_3_ nanoparticles according to this paper is 0.3 g/L and there is no significant difference between distinct sizes, but smaller nanoparticles seem to be less sensitive to various concentrations.

Different results were obtained by Beroual et al. in [102] where the DC-BDV test was examined on the mixture of SE and Al_2_O_3_ nanoparticles. For both particle sizes, 13 and 50 nm, the optimal concentration was 0.05 g/L with enhancement 25.5% and 12.7%, respectively. With higher concentration, the DC-BDV decrement was more significant up to 32.6% at concentration 0.3 g/L. Considering the experiment made by Beroual et al. optimal concentration for nanofluid with Al_2_O_3_ nanoparticles are 0.05 g/L with the size of nanoparticles 13 nm. An overview of the DC-BDV of nanofluids containing Al_2_O_3_ nanoparticles is presented in Table 3.

#### 4.1.3. LI-Breakdown Voltage

Another experiment made by Beroual and Khaled is focused on the examination of lightning breakdown voltage (LI-BDV) of mixture NE (MIDEL 1204) and SE (MIDEL 7131) with Al_2_O_3_ nanoparticles of size 50 nm [103]. There were observable differences between these oils in the enhancement of the BDV. NE showed best results at concentration 0.05 g/L (enhancement 16.76%) and with higher concentration values, the enhancement was lowered to 2.12% at concentration 0.4 g/L. SE based nanofluid showed higher average enhancement and the maximum value was reached at concentration 0.3 g/L (18.32%). These data confirmed that the optimal concentration of nanoparticles is bound to a certain type of oil.

Koutras et al. examined NE Envirotemp FR3 with nanoparticles Al_2_O_3_ of size 30–35 nm and concentration 0.004 wt% [88,104]. AC-BDV enhancement of this combination was 4.1%, but LI-BDV was enhanced by 28.5% under positive polarity, while only 1.5% enhancement was found under negative polarity.

In the mentioned experiment conducted by Rafiq [94], the enhancement of LI-BDV decreased with ageing and after 30 days the value was even lower in comparison to the base fluid. The positive LI-BDV test indicated that nanofluid had higher values than pure oil only at the particular moment of ageing. An overview of the LI-BDV of nanofluids containing Al_2_O_3_ nanoparticles can be seen from Table 4.

#### 4.1.4. PDIV

Partial discharge inception voltage is defined as: “the lowest voltage at which partial discharges are initiated in the test arrangement when the voltage applied to the test object is gradually increased from a lower value at which no such discharges are observed” [105]. In the experiment performed by Koutras et al. [88], the value of PDIV was enhanced by 44% that makes nanofluid less prone to degradation (by discharges) of oil in comparison to the base fluid.

In the mentioned studies carried out by Jacob et al. [98] on the differences in results according to the concentration of surfactant in nanofluid, the PDIV were increased by 1 kV at concentration 0.03 wt% regardless of whether there was a surfactant or no.

Mohamad et al. [106] examined PDIV of nanofluid based on MO and two NE (coconut oil and bleached and deodorized palm oil (RBDPO)) which were modified by surfactant sodium dodecyl sulfate (SDS). Results showed that maximal values of all mixtures were reached at the nanoparticle concentration of 0.001 vol% with SDS. The increment of PDIV in MO, coconut oil and RBDPO was 27%, 39.3%, and 34%, respectively. For comparison, the same samples but without SDS reached enhancement 24.2%, 27.7%, and 23.9%, respectively. Thus, the positive influence of surfactant on the enhancement of PDIV was confirmed. An overview of the PDIV for nanofluids containing Al_2_O_3_ nanoparticles is presented in the following Table 5.

### 4.2. TiO_2_ Nanoparticles

Frequently used nanoparticles for the improvement of insulating oils are semi-conductive TiO_2_ nanoparticles. According to AZO materials [107], the thermal conductivity of TiO_2_ nanoparticles is 11.7 WmK^−1^ at 25 °C, dielectric strength 4 kVmm^−1^ and dissipation factor at 1 MHz is 5 × 10^−4^.

#### 4.2.1. AC-BDV

Olmo et al. [108] examined a mixture of TiO_2_ nanoparticles with NE made from sunflower seeds. The size of nanoparticles was between 10 and 20 nm in diameter. The results of their experiment showed improvement of AC-BDV by 33.2% at concentration 0.5 kg/m^3^ and also other concentrations showed enhancement from 7.6% to 30.4%, although the content of moisture was higher at concentrations 0.1, 0.5, and 1 kg/m^3^. The dissipation factor increased by 13.5% from 0.026 to 0.0295 which is similar to the increment of resistivity by 9.2%. The influence of nanoparticles on heat-transfer properties was not significant. For example, thermal conductivity was unchanged and viscosity rises to 7.2%, but results of the cooling test showed a decrease of 3.9% at concentration 0.5 kg/m^3^, probably caused by thermomagnetic buoyancy forces considering the non-magnetic nature of Titania nanoparticles.

Koutras et al. [109] mixed EnvirotempTM FR3TM NE with TiO_2_ nanoparticles of an average nominal diameter of 21 nm. The mean AC-BDV was enhanced by 22.4% at concentration 0.02 vol% and improvement of dielectric properties were proven at all examined concentrations (0.005–0.04 vol%). LI-BDV of positive charge enhanced its values by 5.2% and 12.9% at concentrations 0.02 vol% and 0.01 vol%, respectively. PDIV value of a paper impregnated with NE was 20% lower than that of a paper impregnated with the nanofluid containing TiO_2_ nanoparticles of concentration 0.02 vol%, which makes a significant difference.

Maneerat et al. [110] examined nanofluids based on NE FR3 and nanoparticles TiO_2_ of diameter less than 100 nm. The experiment described the change in AC-BDV at various temperatures. The enhancement of AC-BDV raised with temperature and at 130 °C and concentration 0.03%, the improvement was 22.8%. At the same concentration, there was a decrement in AC-BDV by 5.5% at temperature 110 °C. All other temperatures and concentrations showed enhancement from 3.5% to 17.5% that confirms that temperature does not affect nanofluids more significantly than pure oils.

Pyrgioti et al. [111] mixed NE FR3 and TiO_2_ nanoparticles of average diameter 21 nm to confirm enhancement of AC-BDV. Results showed that mean AC-BDV of pure NE was 65.6 kV and nanofluid of concentrations 0.004 wt% and 0.02 wt were 68.4 and 69.8 kV, respectively. Therefore, the enhancement of AC-BDV reached 6.4%.

Sun et al. [112] mixed naphthenic transformer oil (25# Karamay) with TiO_2_ nanoparticles of diameter less than 10 nm at one concentration 0.075 vol% and compared the results of different dielectric experiments after ageing. The results showed enhancement at each period of ageing from 6 days of accelerated ageing (the equivalent is 5 years of ageing time) to 36 days of accelerated ageing (that is 30 years of equivalent ageing time). The examined parameters were AC-BDV, LI-BDV, and PDIV. The difference between values rises for AC-BDV from 3.5% after 6 days of accelerated ageing time to 121% after 36 days of accelerated ageing. LI-BDV enhancement on the twelfth day of the accelerated ageing was 46.3% and the lowest enhancement 33.4% was found on the thirty-sixth day, still constituting an excellent improvement. Enhancement of PDIV during the ageing move from 13.4% to 23.8%, so it can be concluded that the nanofluid with TiO_2_ nanoparticles is considerably less affected by the ageing than the pure oil.

Fernández et al. [113] mixed commercially available vegetable oil with TiO_2_ nanoparticles of diameter 45 nm and examined properties during accelerated ageing. Firstly, the AC-BDV test was held to find an optimal concentration. The highest enhancement around 35% was measured at a concentration 0.04 wt% and all of the examined concentrations reported improved values. TiO_2_ nanoparticles improve susceptibility to ageing and at the end of the experiment, particularly after 300 h of accelerated thermal ageing, the difference between the pure oil and the nanofluid was 28%, so revealing that nanofluids are not significantly affected by ageing and they are even less sensible than the pure oil. The dissipation factor of nanofluid is higher than that of pure oil and during ageing, the related curves for pure oil and nanofluid were almost similar, so the difference between them did not change significantly.

Méndez et al. [114] examined a vegetable fluid with addition of TiO_2_ nanoparticles with an average diameter ranging from 10 to 20 nm. Firstly, thermal conductivity was measured and no significant changes were observed at any concentration. AC-BDV showed improvement of dielectric properties at each level of concentration except for the highest one (1 kg/m^3^). Enhancement of AC-BDV was optimal at concentration 0.5 kg/m^3^ where the value reached 33.2%.

Opranti et al. [100] in the mentioned experiment also examined NE with TiO_2_ nanoparticles. The optimal concentration 0.6 wt% showed enhancement in breakdown strength 32% in comparison with pure palm kernel oil. With higher concentration up to 1 wt%, the breakdown strength showed decrement up to 27.5% (1 wt%). Al_2_O_3_ nanofluid showed better or almost the same results for all concentrations of nanofluid that makes it more suitable for high voltage application according to this experiment.

Muangpratoom [115] compared the enhancement of breakdown strength at different temperatures. The nanofluid consisted of a palm oil mixed with TiO_2_ nanoparticles of size 40 nm. Two concentrations were measured 0.01 vol% and 0.03 vol%. Both concentrations showed enhancement at each level of temperature from 35 to 90 °C and breakdown strength increased with temperature. However, the nanofluid with 0.03 vol% of TiO_2_ nanoparticles exhibited better results and the highest enhancement 35.5% was measured at temperature 50 °C.

Gayathri et al. [116] compared a mixture of NE (MIDEL 1204—20% of total volume) and MO (Apar Power oil TO335—80% of total volume) and its variations mixed with nanoparticles TiO_2_ of diameter less than 5 nm and surfactants CTAB and oleic acid. CIV (corona inception voltage) showed the highest enhancement around 45% at concentration 25 mg/L of TiO_2_ nanoparticles in the mixed oil. Various concentrations of CTAB and oleic acid were added to the nanofluid of mentioned concentration and the highest improvement with CTAB (0.5 mg/L) was around 31% and for oleic acid (5 µL/L) around 12%. Thus, if we compare the base fluid and the nanofluid with CTAB as the surfactant, there is about 90% enhancement of CIV. AC-BDV enhancement was around 8% at the same concentration (25 mg/L) and with CTAB of concentration 0.5 mg/L, there was enhancement around 17%, as compared with the base fluid. The nanofluid with oleic acid of concentration 5 µL/L showed enhancement of AC-BDV around 13%. The results of positive DC-BDV are very similar to AC-BDV, but the values are about 1 kV higher than those at AC-BDV. Negative DC-BDV was not influenced by the surfactants and with a higher concentration of CTAB or oleic acid, there was a decrease in DC-BDV. However, the nanofluid of concentration 25 mg/L improved its value by around 12% and raised to above 50 kV, while AC-BDV and positive DC-BDV were found around 25 kV. Other examinations of samples showed excellent stability mainly with CTAB surfactant, flashpoint and thermal conductivity showed marginal improvement in values after addition of TiO_2_ nanoparticles. Viscosity showed negligible changes in exposure to AC and DC BDV that indicated a high stability of the nanofluid. Based on these results, it can be concluded that CTAB is more effective surfactant than oleic acid. An overview of the AC-BDV of nanofluids containing TiO_2_ nanoparticles is presented in the following Table 6.

#### 4.2.2. DC-BDV

Oparanti et al. in their study [90] examined thermophysical properties of nanofluids with TiO_2_ nanoparticles. Kernel oil with TiO_2_ nanoparticles exhibits an improved flash point by 11% (at 1 wt%), however, the pour point increased its value by 37% (at 1 wt%). Viscosity increment was not significant mainly at 40 and 60 °C, but with higher concentration and temperature there were increased values up to 3%. The dielectric loss was reduced from 0.044 to 0.0026, but in comparison with Al_2_O_3_ nanoparticles with a value of 0.0013, it is not so significant. DC-BDV values were very similar if one compares the effect of Al_2_O_3_ and TiO_2_ nanoparticles and the difference between nanofluids with concentration 0.6 wt% were only 1 kV, and enhancement at this concentration was 33.3%. To sum up, the difference between TiO_2_ and Al_2_O_3_ nanofluids were not so significant, but as the result of this experiment, Al_2_O_3_ nanoparticles are considered to be more suitable for the high voltage application.

#### 4.2.3. LI-BDV

Wang et al. [117] examined LI-BDV of positive and negative polarity on a nanofluid made of MO KI25X and Titania nanoparticles of diameter approximately 20 nm. There were different results between the measurement of positive and negative polarity. Positive polarity showed enhancement of LI-BDV for all concentrations of nanoparticles and the highest concentration 0.09808% improved the value of LI-BDV by around 39%. On the other hand, the negative polarity LI-BDV test indicates that nanoparticles affect the properties of the base fluid negatively. Decrement of LI-BDV values was found for each concentration and it moved from around 2.1% to approximately 25%. According to [118], referred by these authors, it is caused by the splitting of electrons propagating in different directions, and branching of streamers in a highly nonuniform electric field when the voltage polarity varies.

#### 4.2.4. Other Thermophysical Properties

Amalanathan et al. [119] examined MIDEL 1215 mixed with CTAB surfactant and TiO_2_ nanoparticles with a size of 5 nm. Significant results of stability proved by Zeta potential showed values above 60 mV at all examined concentrations (25–100 mg/L) and with CTAB the values did not fall under 80 mV. Zeta potential of nanofluid with 50 mg/L of TiO_2_ nanoparticles and 1 mg/L of CTAB reached a value more than 100 mV, and if we take into account that nanofluids with values of Zeta potential greater than 30 mV are considered as stable and greater than 60 mV are considered as extremely stable, this nanofluid had a significant stability. AC Corona inception voltage (CIV) was enhanced by about 75% at concentration 50 mg/L of TiO_2_. Different concentrations of CTAB at a concentration of TiO_2_ 50 mg/L were also examined and results showed that enhancement was from 213–250% in comparison with the pure NE. The dissipation factor of the sample with TiO_2_ nanoparticles and CTAB were more than doubled (at 30 °C) and at 90 °C it was almost tripled in comparison with the value for the pure oil. This result can be considered as a negative impact of the added substances. The measured dynamic viscosity according to the presented results did not show any significant differences between various samples.

Li et al. [120] compared studies of MIDEL 7131 (SE) and Castrol oil (NE) doped with TiO_2_ nanoparticles of an average diameter of 30 nm. The examined viscosity showed that nanofluid with SE had 10 times lower values, so indicating better heat-transfer properties. The main experiment compared values of breakdown strengths (kV/cm) of these two types of nanofluid. For NE, the optimal concentration was 1 vol% where the average breakdown strength was increased by 55% with a value of more than 1000 kV/cm and for SE there was an enhancement of 12% compared with the base fluid with a value of around 900 kV/cm.

Chakraborty et al. [91] prepared nanofluids from mineral oil and NE FR3 and nanoparticles TiO_2_ and Al_2_O_3_ with a diameter from 40 to 80 nm. The results indicated that the dissipation factor is influenced by the concrete combination of the base fluid and the nanoparticles. The dissipation factor increased for combinations of MO with Al_2_O_3_ and NE with TiO_2_ nanoparticles. On the other hand, the combination of MO and TiO_2_ nanoparticles showed a decrement, as well as the combination of NE and Al_2_O_3_ nanoparticles. The importance of examination of a concrete combination is proven by these different results for various combinations of the insulating oil and nanoparticles.

### 4.3. Fe_3_O_4_ and Fe_2_O_3_ Nanoparticles

#### 4.3.1. AC-BDV

Khaled et al. [93] compared results of AC-BDV of SE (MIDEL 7131) with Al_2_O_3_, Fe_3_O_4_, and SiO_2_ nanoparticles. Fe_3_O_4_ nanoparticles of a diameter 50 nm enhanced the AC-BDV up to 47.78% at a concentration 0.4 g/L that is the highest enhancement among all samples. The enhancement 17.83% was at a concentration 0.3 g/L, but samples with a lower concentration (0.05 and 0.2 g/L) exhibited a decreased AC-BDV by 6.06% and 0.05%, respectively.

Mendez et al. in [114] compare nanofluids with TiO_2_ nanoparticles and Fe_2_O_3_ nanoparticles with diameters from 10 to 20 nm. A vegetable fluid was chosen as a base fluid, while the nanoparticle concentrations varied between 0.1 and 0.5 kg/m^3^. Similar results of thermal conductivity were obtained when both added nanoparticles did not manifest significant changes. The enhancement of AC-BDV by 15.1% was at an optimal concentration 0.2 kg/m^3^. Remaining concentrations, 0.1 and 0.3 kg/m^3^, showed an increment in AC-BDV, while higher concentrations, 0.4 and 0.5 kg/m^3^, decreased the value of AC-BDV. In comparison with TiO_2_ nanoparticles with an improvement of 33.2%, the mixture of NE and Fe_3_O_4_ nanoparticles are less effective according to this experiment.

Primo et al. [121] mixed MO Nytro 4000X with Fe_3_O_4_ nanoparticles of diameter 10 nm. Concentrations of nanoparticles were 0.05, 0.1, and 0.2 g/L and enhancements of AC-BDV were 7.03%, 8.16% and 9.41%, respectively.

Hussain et al. [122] prepared nanofluids made of NE and SE with Fe_3_O_4_ nanoparticles of size ranging from 50 to 100 nm. AC-BDV was measured with two electrode systems, mushroom–mushroom (M-M) and sphere–sphere (S-S). The difference between these systems was about 30 kV. The electrode system M-M yielded different results for nanofluids with NE and SE as a base fluid. The highest enhancement 20.7% for nanofluid with SE was at a concentration 0.0022 wt%. On the other hand, NE-based nanofluid showed the highest enhancement 12.1% at a concentration 0.004 wt%. The electrode system S-S confirmed optimal concentrations for nanofluids and the highest enhancements of SE and NE based nanofluids were 30.7% and 33.4%, respectively. The results confirm that each combination of the base fluid and nanoparticles have an optimal concentration that was proved by different kinds of electrode systems in this experiment.

Olmo et al. [123] used Fe_2_O_3_ nanoparticles with a mean diameter between 10 and 20 nm mixed with NE. Six different concentrations from 0.1 to 0.5 kg/m^3^ were used to examine different physical and dielectric properties as viscosity, thermal conductivity and dielectric strength. No influence on the two first was noticed, probably due to the low concentrations of nanoparticles. The optimal concentration of Fe_2_O_3_ nanoparticles 0.2 kg/m^3^ improved AC-BDV by 16%. The nanofluid with 0.1 and 0.3 kg/m^3^ of nanoparticles showed an enhancement too, however, remaining higher concentrations 0.4 and 0.5 kg/m^3^ decreased the value of AC-BDV.

Charalampakos et al. [124] mixed NE Envirotemp FR3 with oleate-coated colloidal magnetic iron oxide nanocrystals (colMIONs). Different concentrations were tested until the value of AC-BDV decreased at a concentration around 0.014 wt%. AC-BDV enhancement was only at 0.008 wt% and 0.012 wt% concentrations, at which the enhancement reaches its maximum value of around 17%. An overview of the AC-BDV of nanofluids containing iron oxide nanoparticles is presented in Table 7.

#### 4.3.2. DC-BDV

Fe_3_O_4_ as conductive nanoparticles are used mainly for the enhancement of dielectric properties. Similarly, for Al_2_O_3_ nanoparticles, Khaled et al. [101] examined NE MIDEL 1204 with Fe_3_O_4_ nanoparticles of diameter 50 nm. Nanofluid with Fe_3_O_4_ showed the highest enhancement (10.56%) among the presented samples (Fe_3_O_4_, Al_2_O_3_, SiO_2_) at concentration 0.2 g/L. However, nanofluids with remaining concentrations (0.05, 0.3, and 0.4 g/L) exhibited decreased values of DC-BDV from 0.62% to 13.84% at a concentration 0.3 g/L. These facts indicate sensitivity to the optimal concentration of Fe_3_O_4_ nanoparticles in nanofluid to reach the improved DC-BDV properties.

A similar experiment made by Beroual et al. [102] with different base fluids combines SE MIDEL 7131 with Fe_3_O_4_ nanoparticles with an average diameter of 50 nm. Enhancement of DC-BDV was observed only at concentration 0.05 g/L with an improvement of 9.8%. Higher concentrations decreased the values of DC-BDV by 7.89%, 1.05%, and 2.03% at concentrations 0.2, 0.3, and 0.4 g/L, respectively.

#### 4.3.3. LI-BDV

Beroual et al. [103] examined also negative LI-BDV on NE (MIDEL 1204) and SE (MIDEL 7131) with different nanoparticles. Fe_3_O_4_ nanoparticles with an average size of 50 nm improved the U_50%_ (a parameter that represents a value of LI-BDV with 50% probability of occurrence according to the normal and Weibull distribution laws) LI-BDV value of SE at each examined concentration. The enhancement decreased with concentration from 25.57% (0.05 g/L) to 6.37% (0.4 g/L). NE as a pure oil had a higher value of LI-BDV and the difference was 2.6%. After the addition of the nanoparticles, NE did not reach values as high as SE. The highest enhancement of NE nanofluid 7.51% was at concentration 0.2 g/L and remaining concentrations 0.05, 0.3, and 0.4 g/L showed an increment (decrement) 2.62%, −4.45%, and −8.29%, respectively. This experiment indicated that SE MIDEL 7131 is more appropriate than NE MIDEL 1204 as the base fluid for the mentioned applications.

Primo et al. [125] made nanofluid by adding Fe_3_O_4_ nanoparticles to MO Nytro 4000X and the size of nanoparticles was 10 nm. Positive and negative LI-BDV tests were examined and an enhancement for positive LI-BDV moved from 3.99% at concentration 0.2 g/L to 49.69% at the highest concentration 0.6 g/L. The nanofluid tested for negative LI-BDV showed an enhancement from 0.5% at concentration 0.6 g/L to 8.81% at concentration 0.4 g/L.

Accelerated thermal ageing tests were performed on nanofluids with superparamagnetic iron oxide nanoparticles by Kurimský et al. [126]. They found that the transformer oil is more resistive to thermal ageing than the nanofluids. The thermally influenced breakdown performance of nanofluids exhibited deteriorative behavior with increasing nanoparticle concentration. Recently, Rajnak et al. [127] showed that the presence of magnetite nanoparticles in transformer oil does not necessarily yield an enhancement in the AC breakdown voltage. They discuss that the nanoparticle surface modification may play a decisive role in the breakdown mechanism. Another study revealed that a small amount of magnetite nanoparticles (0.001 vol%) can remarkably enhance partial discharge activity in a naphthenic oil but not in an oil prepared by a gas-to-liquid technology [128]. Moreover, transformer oil-based nanofluids containing magnetic nanoparticles were applied in prototype transformers. It was reported that the prototype transformers filled with the magnetic fluids exhibited superior performance [20,129]. The enhancement of the cooling efficiency was partially achieved by the developed thermomagnetic convection. On the other hand, the application of an external magnetic field to magnetic nanofluids may control their dielectric response, but also increases the viscosity and thermal conductivity, depending on the particular configuration of the magnetic field and the measuring sensor [130].

### 4.4. SiO_2_ Nanoparticles

#### 4.4.1. AC-BDV

Khaled et al. in the mentioned experiment [93] mixed SE MIDEL 7131 with silica nanoparticles of diameter from 10 to 20 nm. Unlike DC-BDV results, enhancement of AC-BDV moved from 19.17% at concentration 0.05 g/L to 31.5% at a concentration 0.4 g/L, so with the rising concentration, the values of AC-BDV raised simultaneously.

Shill et al. [131] examined mixtures of MO (POWER OIL TO 335X) and SE (MIDEL 7131) with SiO_2_ nanoparticles with an average size of less than 50 nm. Enhancement of AC-BDV of these samples reached only about 2% for MO and about 4% for SE at a concentration 0.02% *w*/*v*. On the other hand, with higher concentration, there is a decrement of around 9% for MO and around 7% for SE. To sum up, results indicate that SiO_2_ nanoparticles are not suitable for high voltage applications. This experiment dealt also with a thermal conductivity that raised with concentration and enhancement was 14% for SE at concentration 0.05% *w*/*v* and around 9% for MO at the same concentration. The viscosity of MO nanofluid was stable at all examined concentrations however, SE nanofluid showed a 12% increment at the highest examined concentration.

Charalampakos et al. [124] prepared a mixture of NE Envirotemp FR3 with silica nanoparticles with an average diameter of 12 nm. The results confirmed that SiO_2_ nanoparticles are not suitable for the enhancement of dielectric properties, as the values of all concentrations showed a decrement in AC-BDV. The decrement moved from around 9% at a concentration 0.02 wt% to around 150% at the highest examined concentration 0.024 wt%.

Baharuddin et al. [99] mixed Hyrax Hypertrans MO with SiO_2_ nanoparticles with an average diameter of 12 nm. The first experiment determined the optimal concentration of nanoparticles and then CTAB surfactant was added to test the properties of nanofluid. Optimal concentration was 0.1 wt% with an average enhancement of 63% and after addition of CTAB surfactant, the values of AC-BDV decreased with increasing CTAB concentration. An overview of the AC-BDV of nanofluids containing SiO_2_ nanoparticles is shown in Table 8.

#### 4.4.2. DC-BDV

Silica nanoparticles are examined to improve the thermal and dielectric properties of transformer oils. Nevertheless, they are insulating kinds of nanoparticles and DC-BDV tests indicate a decrement of dielectric performance of base fluids. Khaled and Beroual et al. in the mentioned experiments [101,102] examined NE (MIDEL 1204) and SE (MIDEL 7131) with SiO_2_ nanoparticles with sizes ranging from 10 to 20 nm. Both of these experiments showed a decrease in DC-BDV, particularly silica nanoparticles in SE decreased the value from 8.07% at a concentration 0.05 g/L to 59.03% at a concentration 0.4 g/L. NE with silica nanoparticles reached similar results when the decrement moved from 5.14% at a concentration 0.2 g/L to 54.6% at a concentration 0.4 g/L. It can be concluded that SiO_2_ nanoparticles are not suitable for high voltage applications, especially when one takes into account the presented DC-BDV values.

#### 4.4.3. LI-BDV

Beroual et al. [103] tested the improvement of negative LI-BDV in SE (MIDEL 7131) and NE (MIDEL 1204) nanofluids with SiO_2_ nanoparticles with a diameter of 50 nm. SE enhancement of U_50%_ LI-BDV moved from 3.5% at the concentration 0.4 g/L to 21.84% at concentration 0.3 g/L. All of the SE samples showed improvement in LI-BDV. NE nanofluid similarly showed an improvement of the values at all concentrations from 7.75% (0.05 g/L) to 13.1% (0.2 g/L), so it can be concluded that silica nanoparticles are suitable to improve the negative LI-BDV.

### 4.5. Graphene Nanoparticles

Almeida et al. [132] mixed graphene nanoparticles with mineral transformer oil to test thermophysical and dielectric properties of the resulting nanofluid. The samples were sorted by the concentrations 0.01 wt%, 0.03 wt%, and 0.05 wt%. The viscosity of nanofluid raised with concentration, particularly, for 0.03 wt% and 0.05 wt% of graphene in the base fluid, the viscosity increased its value by 9.9% and 19.88%, respectively. Dielectric properties were described by electrical conductivity and dissipation factor. The electrical conductivity of nanofluid (0.05 wt% of graphene) was 4.76 times higher than that of the base fluid that may indicate degraded insulating properties. Dissipation factor, as well as the electrical conductivity raised with concentration and the highest measured value was increased more than 10 times, as compared with the base fluid.

Farade et al. [133] prepared a nanofluid made of graphene oxide (GO) and NE (cottonseed oil). AC-BDV of the measured nanofluid exhibited an enhanced value by 25% at a weight percentage of 0.02 wt%. AC-BDV was enhanced also at other concentrations of GO nanoparticles. Moreover, the dissipation factor was reduced at each concentration, so it can be concluded that a combination of GO and cottonseed oil is suitable for the dielectric application. Thermal conductivity as the thermophysical property was also enhanced at each level of concentration and the value raised with concentration up to the enhancement of 36.4% at a concentration 0.05 wt%.

### 4.6. Fullerene (C_60_) Nanoparticles

Szcześniak et al. [134] examined C_60_ nanoparticles with a diameter of approximately 0.7 nm mixed with NE FR3 before and after 164 h of accelerated ageing at temperature 150 °C. As for thermophysical properties, the viscosity of the nanofluid remains almost unchanged even after ageing, so the nanofluid is suitable as a cooling medium as well as a base fluid. AC-BDV of the studied nanofluids was lowered by around 10% and 5% at concentrations 500 and 250 mg/L, respectively, but these results were measured before ageing. After ageing, there was an enhancement of around 23% (when comparing the aged base fluid and the aged nanofluid of concentration 500 mg/L). Dissipation factor raised with concentration of nanoparticles in fluids before and also after the accelerated ageing.

Huang et al. [135] mixed NE obtained from raw rapeseed oil and MO with C_60_ nanoparticles with a diameter from 4 to 6 nm. The dissipation factor was decreased by 20.1% for NE with C_60_ nanoparticles of concentration 100 mg/L and decrement in MO was around 50% at a concentration 50 mg/L. For both nanofluids, a higher concentration means a higher dissipation factor starting from the mentioned concentrations. AC-BDV in NE was improved at concentrations 50–150 mg/L, and at higher concentrations there was a decrement. The enhancement moved from around 2% to around 8%. MO mixed with C_60_ nanoparticles showed a decrement only at a concentration 50 mg/L. The optimal concentration of fullerene for MO is 200 mg/L when the enhancement reached around 21%.

### 4.7. h-BN Nanoparticles

Maharana et al. [136] used insulating and high thermal conductivity-based hexagonal boron nitride (h-BN) nanoparticles of size 50–150 nm to enhance the properties of NE FR3. AC-BDV test showed an enhancement around 5% but with ageing, the enhancement raised to a maximum around 11% at 300 h of accelerated ageing. The nanofluid is less sensitive to ageing in comparison to the base fluid. The dissipation factor was almost unchanged after the addition of h-BN nanoparticles. Moreover, after 500 h of accelerated ageing, the nanofluid had around 21% lower AC-BDV value than the pure NE.

Taha–Tijerina et al. [137] tested h-BN, molybdenum disulfide (MoS_2_) and their combination in SE (MIDEL7131) and NE Envirotemp FR3. The measurements were focused on the enhancement of thermal conductivity. Hybrid composition h-BN/MoS_2_ of the nanofluid was used at a mass ratio of 1:1 (h-BN and MoS_2_). In all cases, the enhancement was increased with the concentration of nanoparticles and the highest enhancement was at a temperature around 323 K. In SE, enhancements were around 24%, 27%, and 31% for h-BN, MoS_2_ and h-BN/MoS_2_, respectively. NE showed almost the same results of enhancement, particularly 20%, 22%, and 31% for h-BN, MoS_2_, and h-BN/MoS_2_, respectively. The hybrid composition had the highest enhancement in both cases even though both of the used nanoparticles have different thermal conductivities.

### 4.8. CCTO Nanoparticles

Thomas et al. [138] undertook study on CCTO (CaCu_3_Ti_4_O_12_) nanoparticles mixed with SE MIDEL7131. Concentrations of CCTO in SE were 0.001–0.05 vol%. As for dielectric properties, AC-BDV, LI-BDV, PDIV, and loss tangent measurements were performed. AC-BDV enhancement moved from 8.33% at the concentration 0.05 vol% to 41.6% at concentration 0.005 vol%. Similar results were in LI-BDV measurements where enhancement moved from 11.2% (0.05 vol%) to 17.3% (0.005 vol%) that indicated the optimal concentration at a level of 0.005 vol%. PDIV enhancement increased with concentration from 10.06% at the lowest concentration to 34.95% at the highest concentration. Loss tangent at 90 °C increased with concentration too. At 0.001 vol% there was an increment 11.86%; however, a decrease at 0.05 vol% was 72.88% that made CCTO suitable for the dielectric property enhancement. Thermophysical properties of CCTO nanofluid indicate that 0.005 vol% is optimal for CCTO application in SE, where increment in viscosity was low (1.7%), the flash point had the highest result (enhancement 18.88%) and thermal conductivity enhancement was 6.89%.

In other work from Thomas et al. [139], CCTO and BCZT (Ba_0.85_Ca_0.15_Zr_0.1_Ti_0.9_O_3_) nanoparticles mixed with SE MIDEL 7131 were examined. The value of AC-BDV was improved for each concentration of both nanoparticles. For CCTO, the enhancement moved from 21.7% at a concentration 0.001 wt% to 41.7% at a concentration 0.005 wt%. The enhancement of AC-BDV for BCZT moved from 6.7% to 20% at the same concentrations of nanoparticles as for CCTO. The dissipation factor at 90 °C raised with concentration for BCZT to 58.3% increment. The dissipation factor for CCTO nanofluid had its peak at a concentration of 0.001 wt% where the value was 10 times higher than that measured for the base fluid. At a concentration 0.005 wt%, the dissipation factor reached a value about 4.4 times higher than in pure SE. As for thermophysical properties, the flash point raised with concentration and maximal enhancement for CCTO was 18.9%, while BCZT decreased the value of the flash point at all concentrations and the highest decrement 9.8% was at a concentration 0.001 wt%. Viscosity did not change significantly and maximal increment was 4.1%. Thermal conductivities of both nanofluids were the same with the maximal enhancement of 30.3% at a concentration 0.005 wt%.

Roy et al. [140] mixed SE USEO with CCTO nanoparticles at concentrations 0.005 vol%, 0.1 vol%, and 0.05 vol%. The results of this experiment showed contrary values in comparison with the mentioned experiments carried out by Thomas et al. [138]. AC-BDV was decreased by 35.8% at the highest examined concentration and by 24.7% at the lowest one. The loss tangent increment at 90 °C moved from 13.5% (0.005 vol%) to 28.7% (0.05 vol%). The viscosity increment was not significant at any temperature with the highest value of 1.07%. The flash point decrement moved from 1.95% (0.05 vol%) to 9.3% at a concentration 0.005 vol%.

### 4.9. ZnO Nanoparticles

Duzkaya et al. [141] used NE MIDEL eN 1204 and ZnO nanoparticles of average diameter around 25 nm to examine the AC-BDV enhancement. At the maximal and minimal concentration, there was a decrement in AC-BDV from 2.7% (0.05 g/L) to 9.46% (0.4 g/L). At concentrations 0.1, 0.2, and 0.3 g/L, there were similar results with the enhancement between 5.94% (0.2 g/L) and 5.2% (0.3 g/L). It can be concluded that the optimal concentration of ZnO nanoparticles in this particular NE is around 0.2 g/L.

Fernández et al. in their experiment [113] compared TiO_2_ nanoparticles with ZnO nanoparticles with a diameter of 60 nm. Both nanofluids have their optimal concentration of 0.04 wt% however, the enhancement of TiO_2_ nanofluid was around 35% while ZnO nanofluid showed enhancement around 29%. During the accelerated ageing, the enhancement of ZnO nanofluid decreased to around 12.5% at 300 h, while TiO_2_ nanofluid reached higher values during the whole accelerated ageing test. While TiO_2_ nanoparticles did not change values of dissipation factor during the ageing significantly, the dissipation factor of ZnO nanofluid after 50 h of accelerated ageing started to rise from the value around 0.13 to around 0.45 at 300 h of ageing that can indicate the unsuitability of ZnO nanoparticles for high voltage applications.

In the reported experiment by Muangpratoom [115], ZnO and BaTiO_3_ nanoparticles of average size 30 and 50 nm were also investigated. Analogous to TiO_2_ nanoparticles, the optimal concentration of the nanoparticles was 0.03% and the highest enhancement was at temperature 50 °C. ZnO showed the highest enhancement among all samples that reached 37.15%. The highest enhancement for BaTiO_3_ nanofluid was 27.7%. All of the measured samples enhanced the breakdown strength at each level of temperature and concentration.

### 4.10. Other Nanoparticles

Koutras et al. [88,104] examined dielectric properties of Al_2_O_3_ and SiC nanoparticles of average diameter between 45–50 nm for SiC. Both nanofluids contained 0.004 wt% of nanoparticles in the base fluid NE Envirotemp FR3. AC-BDV results showed that the enhancement of SiC nanofluid was 16.31% and Al_2_O_3_ nanofluid exhibited 11.76% enhancement. Positive LI-BDV showed that higher enhancement was reached for Al_2_O_3_ nanofluid with a value of 28.47%, while 5.59% enhancement was found for SiC nanofluid. The negative LI-BDV study showed opposite results when enhancement in SiC nanofluid was 3.96% while in Al_2_O_3_ nanofluid it was 1.52%. PDIV enhancement was also higher for SiC (92%) than for Al_2_O_3_ (44%).

Hussain et al. in the reported study [122] also examined iron phosphide (Fe_3_P) and cobalt (Co_3_O_4_) nanoparticles mixed with NE and SE. AC-BDV enhancement of NE with Fe_3_P nanoparticles was 19.3% at a concentration 0.004 wt% (M-M electrode system) and 33.5% at the same concentration (S-S electrode system). The highest enhancement of SE was at a concentration 0.0022 wt% with values 20.2% (M-M electrode system) and 31.4% (S-S electrode system). Co_3_O_4_ nanofluid had maximal values of enhancement at a concentration 0.004 wt% for all samples and electrode systems. The value of AC-BDV in NE was increased by 25.1% (M-M) and 32.6% (S-S). The enhancement of SE nanofluid reached values 15% (M-M) and 16.1% (S-S).

Maneerat et al. [110] compared BDV performance of nanofluids with a base oil NE with TiO_2_ and BaTiO_3_ nanoparticles. BaTiO_3_ nanoparticles of size 100 nm enhanced AC-BDV by around 28% at the concentration of 0.05 vol%. The values changed at different temperatures and at 130 °C the optimal concentration changed to 0.03 vol% with an enhancement of around 10%. In comparison with TiO_2_ nanoparticles, BaTiO_3_ showed higher values in most cases.

Thomas [142] made a mixture of SE and MgO nanoparticles of diameter 65 nm. AC-BDV results indicated that enhancement raised linearly up to 0.005 wt% and thereafter it decreased. The maximum value of enhancement reached around 26%. The dissipation factor at 90 °C increased its value at concentration 0.001 wt% 5 times, as compared with the base fluid. At higher concentrations, the dissipation factor was lowered and stabilized. The stabilized value was 2.5 times higher than the value of pure SE. Viscosity was also found stable at all examined concentrations and temperatures.

## 5. Environmental Impacts of Dielectric Nanofluids

The available research papers on the development of dielectric liquids indicate the tendency to consider the environmental aspects in a greater measure. When considering nanofluids as progressive dielectric media for power transformers, the first step toward the eco-friendlier approach is the use of biodegradable base liquids. However, the environmental impacts of nanofluids depend not only on the base liquids but also on the nanoparticles forming the dispersive phase. It was found that the nanoparticle type, concentration of nanoparticles and the production methods have a significant effect on the associated environmental impacts, as recently discussed in [143]. Clearly, in further research into dielectric nanofluids for high-voltage industry, there must be an intensive discussion of the recycling or recovery of nanoparticles added to the base dielectric liquids after their life cycle. Comprehensive and effective pathways for safe disposal and combating of unwanted or unexpected effects of nanoparticles must be considered in the same way as for all other nanoproducts [144,145].

## 6. Conclusions

Nanofluids are a possible future alternative for high voltage applications that can make heat transfer and insulation more effective. This promising alternative to today’s insulating oils brings opportunities for researchers to find an effective combination of a base oil and nanoparticles with an optimal concentration that will be able to meet the criteria for the implementation. Owing to the greater number of papers that deal with nanofluids properties and examination of optimal concentration for a certain combination, it is necessary to unify the interpretation of the results, mainly the physical unit of concentration for better and easier comparison of results. On the other hand, more detailed nanofluid characterization, such as information on nanoparticle crystal structure (especially for those materials exhibiting polymorphism), would help to reproduce and compare the published results by other researchers. In this review, there was an effort to describe nanofluids preparation, options for determining the stability and finally a description of the latest results in this field, mainly insulating properties of biodegradable insulating oils that were sorted by the used nanoparticles. As each of the applied nanoparticle materials may yield different enhancement of dielectric properties of base liquids, it is difficult to provide an unambiguous clue to the right choice for the nanomaterial. In our opinion, this aspect must be considered and experimentally verified specifically for a particular base liquid and for a particular purpose. There is a need for further research of nanofluids before their implementation into high-voltage engineering practice.

## Figures and Tables

**Table 2:** Al_2_O_3_ AC-BDV overview table. The size of NP refers to the nanoparticle core size, while the optimal concentration indicates the concentration value for which the maximal enhancement of AC-BDV was found.

Base Fluid	Preparation of Nanofluid	Size of NP (nm)	Optimal Concentration	Maximum Enhancement	Reference
MO	Two-step; magnetic stirring, ultrasonication	13	0.05 g/L	76.3%	[92]
MO	Two-step; magnetic stirring, ultrasonication	50	0.3 g/L	69.1%	[92]
SE	Two-step; magnetic stirring, ultrasonication	13	0.05 g/L	34.7%	[93]
SE	Two-step; magnetic stirring, ultrasonication	50	0.3 g/L	25.5%	[93]
MO	Two-step; ultrasonication, mechanical stirring	60	0.02 wt%	4.3%	[89]
NE	Two-step; ultrasonication, mechanical stirring	60	0.02 wt%	8.1%	[89]
NE	Two-step; ultrasonication, magnetic stirring	50	0.004 wt%	4.1%	[88]
MO	Two-step; ultrasonication	-	0.8 g/L	14%	[94]
MO	Two-step;mechanical stirring, ultrasonication	<20	0.03 g/L	8% (relative humidity 10%)	[95]
MO	Two-step; ultrasonication, mechanical stirring	60	0.03 wt%	16%	[98]
MO	Two-step; magnetic stirring, ultrasonication	13	0.1 wt%	55.5%	[99]
NE	Two-step; magnetic stirring	-	0.6 wt%	39.7%	[100]

**Table 3:** Al_2_O_3_ DC-BDV overview table. The size of NP refers to the nanoparticle core size, while the optimal concentration indicates the concentration value for which the maximal enhancement of DC-BDV was found.

Base Fluid	Preparation of Nanofluid	Size of NP (nm)	Optimal Concentration	Maximum Enhancement	Reference
NE	Two-step; stirring	18	0.6 wt%	33%	[90]
NE	Two-step; magnetic stirring, ultrasonication	13	0.3 g/L	9.27%	[101]
NE	Two-step; magnetic stirring, ultrasonication	50	0.3 g/L	7.63%	[101]
SE	Two-step; magnetic stirring, ultrasonication	13	0.05 g/L	25.5%	[102]
SE	Two-step; magnetic stirring, ultrasonication	50	0.05 g/L	12.7%	[102]

**Table 4:** Al_2_O_3_ LI-BDV overview table. The size of NP refers to the nanoparticle core size, while the optimal concentration indicates the concentration value for which the maximal enhancement of LI-BDV was found.

Base Fluid	Preparation of Nanofluid	Size of NP (nm)	Optimal Concentration	Maximum Enhancement	Reference
NE	Two-step; magnetic stirring, ultrasonication	50	0.05 g/L	16.76%	[103]
SE	Two-step; magnetic stirring, ultrasonication	50	0.3 g/L	28.5%	[103]
NE	Two-step; ultrasonication, magnetic stirring	50	0.004 wt%	18.32%	[104]
MO	Two-step; ultrasonication	-	0.8 g/L	6.4%	[94]

**Table 5:** Al_2_O_3_ PDIV overview table. The size of NP refers to the nanoparticle core size, while the optimal concentration indicates the concentration value for which the maximal enhancement of PDIV was found.

Base Fluid	Preparation of Nanofluid	Size of NP (nm)	Optimal Concentration	Maximum Enhancement	Reference
NE	Two-step; magnetic stirring, ultrasonication	-	0.001 wt%	39.3%	[106]
MO	Two-step; magnetic stirring, ultrasonication	-	0.001 wt%	27%	[106]
MO	Two-step;ultrasonication, mechanical stirring	60	0.03 wt%	16.6%	[98]
NE	Two-step; ultrasonication, magnetic stirring	50	0.004 wt%	44%	[88]

**Table 6:** TiO_2_ AC-BDV overview table. The size of NP refers to the nanoparticle core size, while the optimal concentration indicates the concentration value for which the maximal enhancement of AC-BDV was found.

Base Fluid	Preparation of Nanofluid	Size of NP (nm)	Optimal Concentration	Highest Enhancement	Reference
NE	Two-step; magtetic stirring, ultrasonication	10–20	0.5 kg/m^3^	33.2%	[108]
NE	Two-step; ultrasonication, mgnetic stirring	21	0.02 vol%	22.4%	[109]
NE	Two-step; magnetic stirring, ultrasonication	<100	0.03 vol% (temperature 130 °C)	22.8%	[110]
NE	Two-step; ultrasonic storring, ultrasonication	21	0.02 wt%	6.4%	[111]
NE	Two-step; ultrasonic bath	45	0.04 wt%	35%	[113]
NE	Two-step; mechanic stirring, ultrasonication	10–20	0.5 kg/m^3^	33.2%	[114]
NE(20%)/MO(80%)	-	<5	25 mg/L (surfactant (CTAB) 0.5 mg/L)	17%	[116]
NE	Two-step; magnetic stirring,	-	0.6 wt%	32%	[100]
NE	Two-step; mechanic stirring, ultrasonication	40	0.03 vol%	35.5%	[115]

**Table 7:** Fe_3_O_4_ and Fe_2_O_3_ AC-BDV overview table. The size of NP refers to the nanoparticle core size, while the optimal concentration indicates the concentration value for which the maximal enhancement of AC-BDV was found.

Base Fluid	Preparation of Nanofluid	Size of NP (nm)	Optimal Concentration	Highest Enhancement	Reference
SE	Two-step; magnetic stirring, ultrasonication	50	0.4 g/L	47.78%	[93]
NE	Two-step; mechanic stirring, ultrasonication	10.20	0.2 kg/m^3^	15.1%	[114]
MO	Two-step; ultrasonication	10	0.2 g/L	9.41%	[121]
NE	Two-step; magnetic stirring, ultrasonication	50–100	0.004 wt%	33.4%	[122]
SE	Two-step; magnetic stirring, ultrasonication	50–100	0.0022 wt%	30.7%	[122]
NE	Two-step; magnetic stirring, ultrasonication	10.20	0.2 kg/m^3^	16%	[123]
NE	Two-step; ultrasonication	-	0.012 wt%	17%	[124]

**Table 8:** SiO_2_ AC-BDV overview table. The size of NP refers to the nanoparticle core size, while the optimal concentration indicates the concentration value for which the maximal enhancement of AC-BDV was found.

Base Fluid	Preparation of Nanofluid		Size of NP (nm)	Optimal Concentration	Highest Enhancement	Reference
SE	Two-step; magnetic stirring, ultrasonication		10.20	0.4 g/L	31.5%	[93]
MO	Two-step; ultrasonication		50	0.02% *w*/*v*	2%	[131]
SE	Two-step; ultrasonication		50	0.02% *w*/*v*	4%	[131]
NE	Two-step; ultrasonication		12	0.02 wt%	−9%	[124]
MO	Two-step; magnetic stirring, ultrasonication		12	0.1 wt%	63%	[99]
